# Understanding the developmental impact of psychological maltreatment through the lens of the dimensional model of adversity and psychopathology

**DOI:** 10.1080/20008066.2026.2616980

**Published:** 2026-01-27

**Authors:** Meret Sophie Wallimann, Katharina Beck, David Cyrill Lätsch

**Affiliations:** a School of Social Work, Institute of Childhood, Youth and Family, Zurich University of Applied Sciences, Zurich, Switzerland; b Division of Developmental and Personality Psychology, University of Basel, Basel, Switzerland; c Child and Adolescent Psychiatric Research Department, University Psychiatric Clinics Basel, Basel, Switzerland

**Keywords:** Emotional maltreatment, psychological maltreatment, psychological abuse, psychological neglect, emotional neglect, emotion regulation, working memory, cognitive development, adolescents, self-report, dimensional model of adversity and psychopathology, Maltrato psicológico, abuso psicológico, negligencia psicológica, abuso emocional, negligencia emocional, regulación emocional, adolescentes, autoinforme, memoria de trabajo, modelo dimensional de adversidad y psicopatología

## Abstract

**Background:** Psychological maltreatment is linked to negative developmental outcomes in adolescents. It includes psychological abuse (PA) and psychological neglect (PN), which are commonly studied as a pair. This study applied the dimensional model of adversity and psychopathology (DMAP), which conceptualises abuse as a threatening experience involving harm or the threat of harm that primarily impairs social-emotional processing, such as emotion regulation, while it views neglect as deprivation or absence of parental care that predominantly affects cognitive functioning, such as working memory.

**Objective:** In an effort to better understand the harmful nature of psychological maltreatment, this study applies the DMAP to understand whether PA and PN have differential effects paving divergent trajectories to psychopathology, hypothesising that PA predicts dysfunctional emotion regulation, whereas PN impairs working memory.

**Method**: A classroom-based online survey was conducted in Switzerland. The analysis incorporated a sample size of 1,207 adolescents across 82 classes. PA, PN, and dysfunctional emotion regulation were assessed using self-report, and working memory was assessed using a performance task. Hierarchical linear regression models were conducted with controls for co-occurring deprivation, threat, and other individual and family factors (e.g. socioeconomic status, attention deficit hyperactivity disorder) at level 1, and for education level and the trauma load of the school class at level 2.

**Results:** In modelling dysfunctional emotion regulation, PA (*β* = 0.45, *p* < .001) was the strongest predictor, whereas PN had no significant effect. In the working memory model, neither PN nor PA was significantly predictive.

**Conclusion:** These findings partially support the DMAP framework, underscoring psychological abuse as an important factor in dysfunctional emotion regulation and psychopathology.

## HIGHLIGHTS

- Psychological abuse and psychological neglect involve distinct pathways to psychopathology, emphasising the need for differentiated research and intervention.
- Psychological abuse is linked to dysfunctional emotion regulation, potentially increasing the risk for psychopathology.
- Psychological neglect showed no association with working memory, suggesting other factors may influence cognitive development.

## Abstract (es)

**Antecedentes:** El maltrato psicológico se asocia con consecuencias negativas en el desarrollo de los adolescentes. Incluye el abuso psicológico (AP) y la negligencia psicológica (NP), los cuales suelen estudiarse de manera conjunta. Este estudio aplicó el Modelo Dimensional de Adversidad y Psicopatología (DMAP por sus siglas en ingles), el cual conceptualiza el abuso como una experiencia amenazante que implica daño o amenaza de daño y que afecta principalmente el procesamiento socioemocional, como la regulación emocional, mientras que considera la negligencia como una privación o ausencia de cuidado parental, que afecta predominantemente el funcionamiento cognitivo, como la memoria de trabajo.

**Objetivo:** Con el fin de comprender mejor la naturaleza perjudicial del maltrato psicológico, este estudio aplica el DMAP para examinar si el AP y la NP tienen efectos diferenciales que conducen a trayectorias divergentes hacia la psicopatología, planteando la hipótesis de que el AP predice la regulación emocional disfuncional, mientras que la NP deteriora la memoria de trabajo.

**Método:** Se realizó una encuesta en línea aplicada en salas de clases en Suiza. El análisis incorporó una muestra de 1.207 adolescentes pertenecientes a 82 cursos. El AP, la NP y la regulación emocional disfuncional fueron evaluados mediante autoinforme, y la memoria de trabajo fue evaluada mediante una tarea de desempeño. Se realizaron modelos de regresión lineal jerárquica con controles de privación y amenaza concurrentes, así como otros factores individuales y familiares (por ejemplo, nivel socioeconómico, trastorno por déficit de atención e hiperactividad) en el nivel 1, y controles por nivel educativo y carga traumática del curso escolar en el nivel 2.

**Resultados:** En el modelo de regulación emocional disfuncional, el AP (*β* = 0,45, *p* < ,001) fue el predictor más fuerte, mientras que la NP no tuvo un efecto significativo. En el modelo de memoria de trabajo, ni la NP ni el AP resultaron predictores significativos.

**Conclusión:** Estos hallazgos apoyan parcialmente el marco del DMAP, destacando el abuso psicológico como un factor importante en la regulación emocional disfuncional y en la psicopatología.

## 1. Introduction

Psychological maltreatment during childhood and adolescence is prevalent (Stoltenborgh et al., 2015), and it is related to a range of negative mental health outcomes (Xiao et al., 2023). Both childhood and adolescence are critical periods during which psychological maltreatment can increase the risk of developing externalising problems (Schlensog-Schuster et al., 2024) and internalising symptoms, such as depression (Humphreys et al., 2020). Psychological maltreatment encompasses the two domains of psychological abuse (PA) and psychological neglect (PN), which are often combined in theory (Glaser, 2002; Slep et al., 2022) and research (Tonmyr et al., 2011). This may be due to their inherent similarity or also to the difficulty of distinguishing PA and PN and discerning whether parental behaviour is inherently psychologically neglectful or abusive. As PA and PN also often co-occur (Mills et al., 2015), this further complicates efforts to differentiate them. So, while the potential harm of psychological maltreatment is well recognised, little is known about whether or how these two domains contribute to that harm.

The dimensional model of adversity and psychopathology (DMAP; McLaughlin & Sheridan, 2016; Sheridan & McLaughlin, 2014), which aims to explain the developmental consequences of childhood adversity, argues that threatening experiences, as exemplified by PA, and depriving environments, as exemplified by PN, exert distinct influences on development. This perspective moves away from viewing adversities as merely singular or cumulative events by focusing on their *core features*, as these have explanatory power regarding how the child is affected (McLaughlin et al., 2021). Such core features can therefore account for differing paths of developmental outcomes, which are linked to distinct risk profiles for psychopathology. More specifically, based on neuroanatomical and functional findings, the DMAP suggests that threat experiences are related to deficits in automatic emotion regulation and heightened emotional reactivity (Lambert et al., 2017; Machlin et al., 2019; Sheridan et al., 2017), which is related to disrupted fear learning, increased threat detection and enhanced vigilance in unsafe environments. In contrast, deprivation is related, in the DMAP, to reduced cognitive control as well as decreased problem-solving and verbal abilities (Lambert et al., 2017; Machlin et al., 2019; Sheridan et al., 2017), as adequate opportunities for learning or social stimulation are lacking. This model can guide the disentanglement of PA and PN by examining whether they are associated with distinct developmental features.

Studies on the harmful effects of maltreatment experiences routinely use emotion regulation, an important transdiagnostic risk factor for psychopathology (Rogier & Velotti, 2023), as the outcome of interest (Gruhn & Compas, 2020). In studies differentiating between PA and PN, PA has shown a stronger association with *increased dysfunctional* emotion regulation (Mills et al., 2015), such as rumination (O’Mahen et al., 2015), than PN, which is in alignment with the DMAP. Studies focusing exclusively on PA have revealed associations with expressive suppression (Thomassin et al., 2016) and avoidant coping (Gagné & Melançon, 2013). Psychologically abused children may use these dysfunctional strategies as protective mechanisms to avoid emotionally negative situations or to adapt to a threatening environment. In contrast, PN has been related to *decreased functional* emotion regulation strategies. This lack of use of adaptive strategies (Mills et al., 2015), such as reappraisal (Hong et al., 2018), is presumably due to a lack of interaction and response to emotional states, resulting in fewer opportunities to learn adaptive strategies.

Focusing exclusively on dysfunctional vs. functional emotion regulation as a sole outcome to better understand and differentiate the effects of PA and PN has two major limitations. First, there is a certain arbitrariness in the distinction between functional and dysfunctional emotion regulation, which has been introduced as the fallacy of uniform efficacy (Bonanno & Burton, 2013). This means that we cannot differentiate emotion regulation into functional and dysfunctional categories without considering the context: Adolescents in unsafe homes may use strategies, such as suppression or heightened reactivity to minimise threat or support rapid responses. Only by persisting in a safe environment, such strategies lose their function and become dysfunctional. Second, PN extends beyond unresponsiveness to emotional states (Slep et al., 2022), also encompassing a lacking support of the child’s social adaptation, such as failure to provide cognitive stimulation or opportunities for learning (Glaser, 2002). As a consequence, PN may impact adolescents beyond deficits in functional emotion regulation, which has been the primary focus of existing research. This limits our understanding of how PN contributes to developmental outcomes and whether it poses a distinct risk for psychopathology.

Working memory has been discussed as a transdiagnostic outcome, particularly in relation to externalising disorders (Huang-Pollock et al., 2017), but findings on the influence of maltreatment on working memory have not been consistent: Early deprivation, including both physical neglect and PN but not threats or abuse, was related to poorer working memory in longitudinal studies in at-risk populations (Awada et al., 2025; Demeusy et al., 2018) and in a sample of institutionalised children (Hanson et al., 2013). In contrast, one retrospective study in an adult clinical sample did not find any association with working memory for any type of maltreatment (Tjoelker et al., 2022). One similar study found an association with physical neglect only (Mørkved et al., 2020) and one found an association with emotional and physical abuse, but not neglect (Miskowiak et al., 2023).

In short, the studies that have shed light on the potential differential effects of PA and PN have primarily focused on *more dysfunctional* vs. *less functional* emotion regulation, and no prior study has aimed to disentangle PA and PN using both measures of dysfunctional emotion regulation and working memory as developmental correlates. Studies have also often fallen short in recognising relevant covariates. Since both PA and PN often occur in a broader and chronic net of poly-adversity (Hodgdon et al., 2018), isolating their effects requires accounting for co-occurring experiences of adversity and the chronicity of exposure to adversity, as these likely impact the measured outcomes (Cowell et al., 2015; Milojevich et al., 2020). Although age and gender are often included, other personal characteristics are equally relevant. One potential factor is the condition attention deficit hyperactivity disorder (ADHD), which is associated with difficulties in emotion regulation and working memory. If not considered, its effects may be wrongly attributed to maltreatment. In studies on adolescents, controlling for school class accounts for adolescents’ intermediate environment, as class-level trauma exposure, the educational level of the class, and school climate may shape functioning and well-being (Day et al., 2017). Having a high number of students burdened by adversity within one school class may adversely affect peer dynamics and learning climate, with implications for both emotion regulation and working memory.

## 2. Aims

This study examined two distinct developmental correlates of PA and PN in a large sample of adolescents from different schools. Considering these two domains of psychological maltreatment in the context of poly-adversity, the study investigated their links with dysfunctional emotion regulation and working memory. Due to the nested structure of the data, hierarchical linear models were employed. The aim was to assess whether the observed patterns aligned with the DMAP, offering insights into the distinct mechanisms through which PA and PN may impede adolescents’ development and act as risk factors for psychopathology.

## 3. Method

### 3.1. Procedure and sample

The study, consisting of an online survey, was conducted in 98 seventh- and ninth-grade classes at 33 schools. These were selected using a stratified sampling approach designed to be representative of a Swiss canton. For this, the target number of schools per district was determined through proportional allocation based on the population of school-age children. Subsequently, the two most common sociodemographic types of municipalities within each district were identified (Bayard et al., 2022). From these resulting lists, municipalities and, within them, public schools (if there were multiple) were randomly selected.

In a first step, school principals of selected schools were asked for participation. Then, caregivers and students were informed via separate letters two weeks before the study. On the day of the online survey, all students provided informed written consent. Parental (passive) consent was sought only from caregivers for seventh- grade students, arguing that ninth-grade students are capable of giving their own consent (Mathews, 2023). Any students who opted out of or withdrew from the online survey were given alternative tasks to complete on the same devices, ensuring that their non-participation could not be detected. Also, the school social worker was informed about the study, and flyers detailing help-seeking resources were distributed. Approval for the study was obtained from the university's Institutional Review Board and the relevant cantonal authorities. The target population and intended sociodemographic distribution were successfully achieved. However, as only 26% of principals agreed to participate, a potential self-selection bias cannot be ruled out. Participation rates were similar (ranging from 22% to 32%) across regional demographics, with the exception of schools in rural and wealthy areas, where only 7% agreed to participate. The initial sample included 1,727 students. Fifty (2.90%) students decided not to participate and 19 (2.28% of the seventh graders) were not able to participate due to a lack of consent from their legal guardian. Of the 1,658 participating students, 1,444 (87.09%) completed the questionnaires. Of those, 67 (4.63%) were excluded due to suspected response bias, including long strings of identical answers that were not logically justifiable (e.g. because of a mix of inverted and non-inverted items)  – especially on the Difficulties in Emotion Regulation Scale, which was the primary outcome –, as well as implausibly fast response times (under 3 s for sentence-long items and under 1.5 s for shorter items) and signs of minimising family problems. This left a sample of *N* = 1,377 cases for the analyses.

### 3.2. Measurement of predictors

#### 3.2.1. Psychological maltreatment

Psychological maltreatment was measured using nine subscales with three to six items (Table 1) that map onto the different dimensions of psychological maltreatment. This selection of subscales was guided by Slep et al.’s (2022) definition of psychological maltreatment and is explained in more detail in Wallimann and Lätsch (2025). The subscales were conceptually grouped as acts of either commission (PA) or omission (PN) based on whether their corresponding items were aligned with active harm or a lack of care (see Table s1, Supplementary). The subscales Terrorizing/Spurning, Isolating, Corrupting, Demanding/Rigid, Emotional Responsiveness (inverted scale), and Abandonment were taken from the Computer Assisted Maltreatment Inventory (CAMI; DiLillo et al., 2010; Nash et al., 2012). The subscale Psychological Control was taken from the Psychological Control Scale-Youth Self-Report (PCS-YSR; Barber, 1996), Overcontrol from the expanded Childhood Trauma Questionnaire (CTQ-33; Şar et al., 2021), and Parental Knowledge (inverted scale) from the Parental Monitoring Scale (Kerr & Stattin, 2000). For each subscale, adolescents were asked to rate their parents’ behaviour from 1 (*strongly disagree*) to 5 (*strongly agree*).

**Table 1.** Subscales of PA and PN.

Subscale	Content of items	
Terrorizing/Spurning	*Hostility, shaming, threatening violence*	**Psychological abuse (PA)**
Psychological Control	*Rejection through love withdrawal*	
Isolating	*Confinement, preventing access to basic needs*	
Overcontrol	*Restrictions, excessive discipline*	
Corrupting	*Modelling/encouraging antisocial behaviour*	
Demanding/Rigid	*Forcing child to live parents’ dreams, meet the needs and expectations of caregiver*	
Emotional Responsiveness (i)	*Unresponsive, lack of affection*	**Psychological neglect (PN)**
Abandonment	*Uninvolved by no supervision, failure to provide care*	
Parental Knowledge (i)	*Knowledge of friends, activities and whereabouts*	

Note: (i) refers to an inverted scale.

The predictor variable PA was computed by aggregating all the item scores from the PA subscales and subsequently *z*-standardising the total score (*α* = 0.93). Similarly, PN was calculated by first inverting the items of the Parental Knowledge and Emotional Responsiveness scale, then summing all the items from the three designated subscales and *z*-standardising the total score (*α* = 0.88). To assess convergent validity, participants answered two self-attribution questions for each construct (PA: whether they felt hurt or bad about themselves due to their parents, and whether parents were harsher or less accepting than others; PN: whether they felt hurt or bad because parents were less caring, and whether parents were less caring and involved than others). PA correlated strongly with its self-attribution items, *r* = .59, 95% CI [.55, .62], *p* < .001. PN also showed a substantial correlation, *r* = .53, 95% CI [.49, .57], *p* < .001. These findings indicate considerable, though not complete, overlap between item-based sum scores and self-attributed experiences.

#### 3.2.2. Adversity and chronicity

Other types of adversity including other threatening experiences like physical abuse, intrafamilial sexual abuse (SA), or witnessing domestic violence (IPV) were measured with a modified version of the CAMI. Each type of threat was assessed using four to five dichotomous items, with 1 indicating that the experience had occurred. To determine the predictor variables, the sum scores of each set of items were compiled and standardised for each type.

To measure additional deprivation, the physical neglect of basic needs like cleanliness was measured with items of the CAMI. To determine the predictor variable physical neglect, the sum score of all the items was computed and standardised. Further, a predictor for household dysfunction and impaired caregiving conditions was computed, both derived from three dichotomous items from the Adverse Childhood Experiences International Questionnaire (ACE-IQ; World Health Organization, 2012): household substance abuse, mental illness, and suicidality. To compute household dysfunction, responses to all three items were summed regardless of which household member was affected, and the resulting score was z-standardised. To compute impaired caregiving conditions, only items where the student indicated that a caregiver was affected were summed, and the resulting score was z-standardised. To assess the timing of psychological maltreatment for each subscale of PA and PN, students were asked to indicate when it had occurred. Specifically, if they scored >2 on any item of the subscales, they were asked (for the entire subscale) if any of the maltreatment had occurred in the past 12 months or prior to that. If it had occurred prior to that, they were asked to indicate in which of the following three time periods the event had occurred: secondary school, primary school, or prior to primary school. The dichotomous variable chronicity indicates whether at least one instance of PA or PN occurred in each time period.

#### 3.2.3. Additionally measured covariates

To measure SES, 20 dichotomous and polytomous household-possession items from the PISA 2018 Home Possessions Scale (Organisation for Economic Co-operation and Development, 2020) were used as a proxy. A generalised partial credit mode was used to extract the factor score, which was then *z*-transformed. DMAP models regularly use low SES as a proxy for deprivation. However, we did not include this SES measure in our deprivation score. In order to conceptually fit into the deprivation score, a clear threshold to identify low-resource households would be needed, which includes information on parental educational attainment or occupational status (Avvisati & Wuyts, 2024). As in this study, SES was only approximated by household possessions; this was considered insufficient to compute a low SES variable. For ADHD, students were asked to report any existing mental, behavioural, or neurodevelopmental disorders from a provided list. If either ADHD or attention deficit disorder (ADD) was selected, the student was coded as having ADHD. Gender was assessed with the item ‘Are you male or female?’ with three response options: Male, female, other (non-binary). All students selecting ‘female’ were coded as female gender, and all identifying as ‘male’ and ‘other’ were grouped into the same reference category, to ensure inclusion of non-binary students.

#### 3.2.4. Level 2 predictors (class level)

To examine between-class variance of outcomes in more detail, two level 2 predictors were computed. For each student, an individual sum trauma score was computed reflecting the number of different types of adversity experienced. This score was based on the presence of PA, PN, physical abuse, intrafamilial SA, IPV, physical neglect, and household dysfunction. Following this, a class-level trauma load variable was calculated by averaging individual trauma scores within each class. The class-level variable lower education was based on the typology of the Swiss secondary education system, which differentiates between four educational paths based on academic performance and career orientation: (1) Higher secondary, (2) pre-higher-secondary-school (more academically oriented tracks) and (3) general or (4) basic schools (more vocationally oriented). A dichotomous predictor was computed in which classes belonging to general or basic schools or sections were categorised as lower educational level, while classes in higher secondary schools or pre-higher-secondary-school sections were coded as the reference category. Importantly, lower educational level primarily reflects reduced academic requirements and does not reflect limited resources and lack of support programmes within schools. Instead, these classes are typically smaller (better teacher-student ratio), and most often include support resources.

### 3.3. Measurement of outcomes

#### 3.3.1. Working memory

Working memory was assessed using a classroom-based (group) assessment, which has been shown to relate to scores in individual assessments (Obradović et al., 2018). There is some evidence that classroom-based assessments (and therefore real-life learning situations) may even provide a more accurate measure of academic achievement (Friso-van den Bos & van de Weijer-Bergsma, 2020). The spatial-span task (SSP) used to measure visuospatial working memory has been designed as a digitalised version of the well-established Corsi block-tapping task (Milner, 1971). Research has shown that there are no significant differences between traditional and digital versions of the task (Arce & McMullen, 2021). For this study, only the backward spatial span was used. The procedure can be described as follows: In each trial, nine randomly located pink squares were spread across a white background. After 500 ms, the first square changed into yellow (for 500 ms) and then turned pink again. After 500 ms, the second square changed into yellow (for 500 ms) and then turned pink again (two-span trial). The participant had to remember which squares had changed colour in the exact order. After a 1000 ms waiting period, the participant had to touch the squares that had changed colour in reverse order (which is signalled by a ‘Go!’ in red colour). The task difficulty increased by one square at a time as the span length increased, ranging from two squares (two-span trial) to nine squares (nine-span trial). For each level of difficulty, participants had three attempts to repeat the sequence of squares. A trial was completed correctly if all the squares were touched in the reverse order of what was shown, and they proceeded to the next level after a correct response. The trial was terminated after three incorrect responses at a given difficulty level. As the backward spatial span was administered in a classroom setting, each participant was guided through the instruction with text, pictures, and sample videos. Three trial tasks with two squares to remember were presented, with feedback after each trial. If all three trials were unsuccessful, the participant had the option of rereading the instructions. The outcome measure of the SSP was the span length (maximum number of squares correctly remembered). Before standardising, differences in span length were examined across gender and grade. While no significant differences were found between genders, seventh- and ninth-grade students differed significantly, *t*(1203.5) = −2.68, *p* < .007; thus, the span length was z-standardised within each grade. The ICC for school class was .11, 95% CI [0.07, 0.16].

#### 3.3.2. Dysfunctional emotion regulation

Dysfunctional emotion regulation was measured using the validated German version of the Difficulties in Emotion Regulation Scale Short Form (DERS-SF; Ehring et al., 2013; Gutzweiler & In-Albon, 2018). The scale consists of six subscales that are measured with three items each, namely, Difficulties Engaging in Goal-Directed Behaviour, Nonacceptance of Emotional Responses, Impulse Control Difficulties, Limited Access to Emotion-Regulation Strategies, Lack of Emotional Awareness (inverted scale), and Lack of Emotional Clarity*.* A confirmatory factor analysis confirmed the scale’s validity with adequate fit: *χ*^2^(120) = 430.001, *p* < .001, CFI = .964, and RMSEA = 0.046. The Lack of Emotional Awareness (inverted) subscale was excluded due to weak correlations with other factors (only significantly associated with impulsivity *r* = .09, *p* = .015). This improved the model fit (*χ*^2^(80) = 289.387) as indicated by higher fit indices (CFI = .971, RMSEA = 0.046). The resulting five-subscale version of the DERS-SF demonstrated good internal consistency (*α* = 0.93) and was used to compute a total score based on all the remaining items. Before standardisation, scalar measurement invariance across grades was established, indicating equivalence in factor structure and item intercepts. However, invariance was not supported across genders. Therefore, total scores were z-standardised separately by gender (female/non-female). The ICC for school class was .03, 95% CI [0.01, 0.07].

### 3.4. Data analysis

The analytic sample originally included 1,377 cases but was reduced due to missing values on the working-memory task (*n* = 4, 0.29%), then due to alterations or technical problems (*n* = 60, 4.36%), and lastly due to missing values on a scale (*n* = 63; 4.57%) or not clearly assignable to a class (*n* = 5, 0.36%). To ensure a robust estimation of the random effects, five school classes with fewer than five students were excluded (*n* = 16; 1.16%). As the working-memory task was assessed in a naturalistic classroom setting, which has been shown to correspond to performance in individual assessments (Obradović et al., 2018), students were required to learn the task independently. However, 23 (1.67%) students did not initiate the task correctly and were therefore excluded from the study, as this did not represent nonfunctional working memory but likely other issues such as a language barrier. As a result, we had a final analytic sample of *n* = 1,207 in 82 classes. We then calculated descriptive statistics on the sample characteristics and predictors. For all the subsequent analyses, we performed hierarchical linear regression analysis (HLM) using the lme4 package, accounting for the nested data structure of the students (level 1) within school classes (level 2). The linearity of the fixed effects was confirmed through inspection of bivariate scatterplots, and multicollinearity was not a concern, all variance-inflation factors being below 3. Homoscedasticity was visually checked by plotting the residuals against fitted values (level 1) and comparing the residuals across the clusters (level 2). Both checks did not indicate any substantial violations.

The normality and independence of the residuals were visually assessed using Q – Q plots and residual-vs.-predicted plots generated with the DHARMa package (Hartig, 2024). Mild deviations from normality were observed at level 1 for the emotion regulation outcome but not for working memory. Similarly, a slightly nonrandom structure was detected in the residuals for emotion regulation but not for working memory. No transformations were applied, as fixed-effect estimates in linear mixed-effects models are generally robust to such assumption violations (Schielzeth et al., 2020). Potential outliers were examined at both the individual and the class levels. No exclusions were made, as no single unit exerted an undue influence on the model estimates, and all the groups met a minimum cell size of five. All the analyses were conducted in R (version 4.4.3).

To validate the DMAP in our data, DMAP models were tested using threat and deprivation scores as predictors for dysfunctional emotion regulation and working memory. For this, deprivation (physical neglect, impaired caregiving conditions, PN) and threat (sexual abuse, physical abuse, witnessing domestic violence, PA) were examined for similar distribution and then combined into z-standardised threat and deprivation scores. Further, reduced scores were calculated, excluding PN (for deprivation) and PA (for threat). These models tested the hypothesised pathways, specifically whether threat is more strongly associated with dysfunctional emotion regulation and whether deprivation is more strongly associated with working memory. To explore the incremental explanatory value of psychological maltreatment, DMAP_red_ (threat without PA, deprivation without PN) was computed. All models were controlled for age, female gender, SES, household dysfunction, ADHD (on level 1), and educational level and trauma load (on level 2).

Subsequently, to parcel out the individual effects of PA and PN, it is hypothesised that PA is more strongly associated with dysfunctional emotion regulation than PN (H1), and PN is more strongly associated with deficits in working memory than PA (H2). To ensure the robustness, a full model including covariates on deprivation, threat, household dysfunction, SES, chronicity, age, and female gender (on level 1) and educational level and trauma load (on level 2) was computed to test if the effect of PA on dysfunctional emotion regulation (H3) and the effect of PN on deficits in working memory (H4) remain significant after accounting for these covariates. While on level 1, predictors of threat and deprivation were added individually in the model, on level 2, they were combined into an overall trauma load, as on level 2, no adversity specific effects were examined. To test H1 and H2, two hierarchical linear models (intercept-only), PA-only and PN-only, were compared regarding AIC and *R*^2^; both controlled for age and female gender. To test H3, PA-only was compared to a full model, which included all the covariates (threat and deprivation). To test H4, PN-only was compared to the full model, which included all the covariates (threat and deprivation). All the models were estimated using maximum likelihood to enable the comparison of fixed effects. Model fit was evaluated using *R*^2^ and likelihood ratio tests. A significance threshold of *p* < .05 was applied for all the statistical tests.

### 3.5. Sensitivity analysis

In the main analysis, PA and PN were included as standardised sum scores (of all subscales). To verify the robustness of the findings, the full models were re-estimated using latent-factor scores for PA and PN derived from a second-order CFA (Wallimann & Lätsch, 2025) and adapted to this sample.

## 4. Results

### 4.1. Sample characteristics and descriptives of predictors

**Figure 1.**
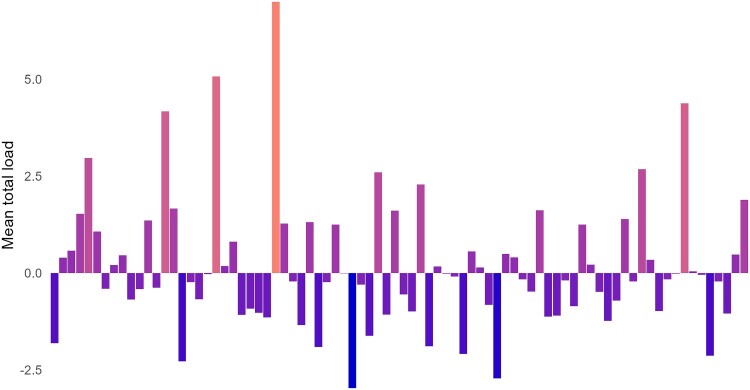
Trauma load (mean total load) differences between school classes. Note: Each bar represents the standardised mean trauma load in every school class. Orange bars show school classes that are above the average trauma load and blue bars show classes with a lower mean trauma load.

The analytic sample comprised *n* = 1,207 students from 82 school classes, with an average class size of 14.72 students. Of these, 546 were in the seventh grade (mean age: 13.2, *SD*: 0.6) with 279 (51.1%) male students, 264 (48.4%) female students, and 3 (0.5%) nonbinary students. Further, 661 were in the ninth grade (mean age: 15.4, *SD*: 0.7) with 294 (44.5%) male students, 362 (54.8%) female students, and 5 (0.8%) nonbinary students. Table 2 summarises the descriptives of the level 1 predictors. Further, 25.19% of students reported that either they or both of their parents had migrated from a low-income country. As visualised in Figure 1, school classes had a high variance in their mean trauma load. Regarding educational level, 225 (18.64%) students had a lower educational level. The correlations of all the predictors are listed in Table s2 in the Supplementary.

**Table 2.** Descriptive statistics on predictors.

Dichotomous Predictors	*n*	%		
Female gender	626	51.86	*** ***	
Chronicity of PA and/or PN	243	20.13		
ADHD	135	11.18		
**Adversity Predictors (theoretical range)^a^**	**Mean**	***SD***	**Min**	**Max**
Sum score PA (0–79)	12.79	14.90	0.00	79.00
Sum score for physical abuse (0–5)	0.54	0.87	0.00	5.00
Sum score intrafamilial SA (0–4)	0.04	0.27	0.00	4.00
Sum score IPV (0–4)	0.58	0.88	0.00	4.00
Sum score PN (0–47)	8.00	8.23	0.00	47.00
Sum score for physical neglect (0–24)	1.26	2.95	0.00	24.00
Sum score of impaired caregiving conditions* (0-3)	0.15	0.43	0.00	3.00
Sum score of household dysfunction (0–3)	0.30	0.62	0.00	3.00
**Continuous Predictors**				
SES^b^	0.01	0.99	−3.69	3.22
Age in years	14.39	1.26	11.67	18.00
Age in months	173.13	14.80	140	216

Note: ^a^For better interpretability, raw scores are presented, ^b^z-standardised, *only included in the deprivation score, but not as individual level 1 predictor. PA: psychological abuse; PN: psychological neglect; SA: sexual abuse; IPV: witnessing domestic violence.

#### 4.2. DMAP and DMAP_red_ models

**Figure 2.**
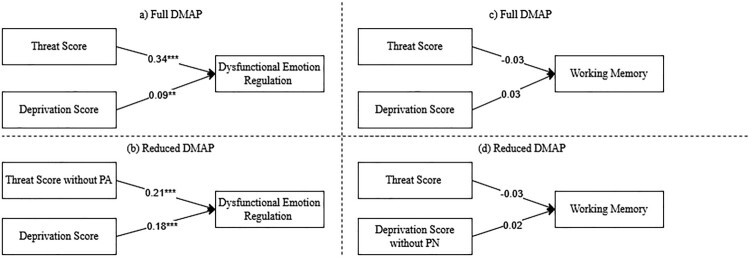
DMAP and DMAP_red_ on dysfunctional emotion regulation and working memory. Note: Models were additionally controlled for age, female gender, SES, household dysfunction, ADHD and lower education [2], and trauma load [2] on the second level (for all statistics, see Tables s10 and s11, Supplementary). *p<.05. **p<.01. ***p<.001.

As visualised in Figure 2, the DMAP model (a) confirmed the effect of threat on dysfunctional emotion regulation, while deprivation was also significantly, but considerably weaker, associated. In the *DMAP_red_* model (b), the effect of threat remained significant but not stronger than that of deprivation. DMAP showed higher explained variance (*R*^2^ = .26) than DMAP_red_ (*R*^2^ = .22). Regarding the covariates, ADHD showed the largest effect (*β* = 0.39 and *β* = 0.43 in models (a) and (b), respectively). Also visualised in Figure 2 on the right, models (c) and (d) did not confirm the effect of deprivation on working memory (both *R*^2^ = .04) with SES (*β* = 0.6) and lower education [2](*β* = 0.44/ 0.43 in Models (c)/(d)) as significant predictors.

#### 4.3. Dysfunctional emotion regulation (H1, H3)

In the PA-only model, PA was a significant positive predictor (*β* = 0.54, *p* < .001). In the PN-only model, PN was likewise a significant predictor (*β* = 0.38, *p* < .001). Model fit was notably better for the PA-only model compared to the PN-only model, with a lower AIC (ΔAIC = −233.5) and higher explained variance (marginal *R*^2^ = .30 vs. .15). The ICC was low (< .04) in both models. The full model (see Table 3) showed a better fit than the PA-only model, with *χ*^2^(11) = 50.79, *p* < .001 (for all the statistics including log-likelihoods, see Supplementary, Table s3), significant covariates include IPV, household dysfunction, female gender, and ADHD.

**Table 3.** Full model on dysfunctional emotion regulation.

Predictors	*β*	95% CI	*p*
*(Intercept)*	0.15	−0.04, 0.35	.127
*PA*	0.45	0.38, 0.53	**<.001**
*PN*	0.03	−0.04, 0.10	.418
*Physical abuse*	−0.04	−0.10, 0.02	.218
*Intrafamilial SA*	−0.02	−0.07, 0.03	.363
*Physical neglect*	−0.02	−0.07, 0.04	.524
*IPV*	0.07	0.01, 0.12	**.024**
*Household dysfunction*	0.08	0.03, 0.13	**.004**
*Age (months)*	−0.03	−0.08, 0.03	.320
*Female gender*	−0.10	−0.19, −0.00	**.045**
*Chronicity of PA and/or PN*	0.07	−0.07, 0.21	.318
*SES*	0.03	−0.02, 0.08	.201
*ADHD*	0.32	0.17, 0.47	**<.001**
*Lower education [2]*	−0.13	−0.28, 0.02	.080
*Trauma load [2]*	0.04	0.00, 0.08	.057
**Log-Likelihood**	−1472.3		
**ICC**	.01		
**AIC**	2978.6		
**Marginal *R*^2^ / conditional *R*^2^**	.326 / .335		

Note: [2] denotes level 2 predictors. PA: psychological abuse; PN: psychological neglect; SA: sexual abuse; IPV: witnessing domestic violence.

**Figure 3.**
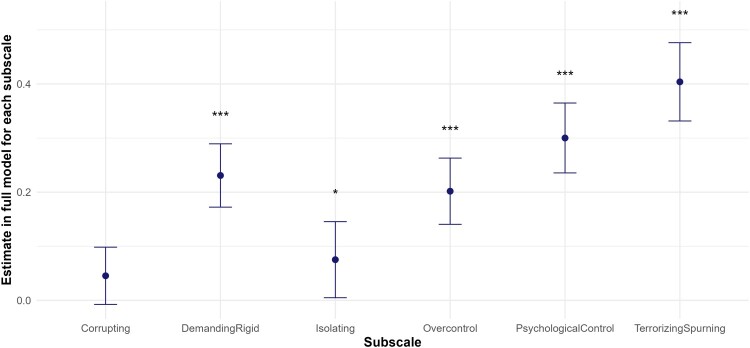
Beta estimates of PA subscales on dysfunctional emotion regulation (95% CI). Note: *p<.05. **p<.01. ***p<.001.

As visualised in Figure 3, in an exploratory analysis, six full models were computed in which PA was replaced by one of the six subscales. This made it possible to compare their predictive values. All the subscales except Corrupting emerged as significant predictors of dysfunctional emotion regulation. The significant standardised estimates ranged from *β* = 0.08 (Isolating) to *β* = 0.40 (Terrorizing/Spurning). To assess their unique contributions, a seventh full model including all the subscales was computed, and their partial *R*^2^ values were compared. The unique variance explained by each subscale was small, and the confidence intervals included zero in all cases. Descriptive statistics for each PA subscale (see Table s5), model summaries (Tables s6, s7), fit statistics (Table s8) and Partial *R*^2^ (Table s9) are reported in the Supplementary.

#### 4.4. Working memory (H2, H4)

In the PN-only model, PN was not significantly associated with working memory. In the PA-only model, PA also showed no significant effect. Model fit was nearly identical (ΔAIC = 0.3), and both models explained minimal variance (marginal *R*^2^ < 0.01). The intraclass correlation (ICC) was .07 in both models. The full model (see Table 4) showed a better fit than the PN-only model, *χ*^2^(11) = 27.57, *p* = .004 (for all statistics including log-likelihoods, see Supplementary, Table s4), significant covariates include SES and lower education [2].

**Table 4.** Full model on working memory.

Predictors	*β*	95% CI	*p*
*(Intercept)*	0.56	0.30, 0.83	**<**.**001**
*PA*	−0.01	−0.10, 0.09	.913
*PN*	0.03	−0.06, 0.11	.555
*Physical abuse*	−0.03	−0.11, 0.04	.375
*Intrafamilial SA*	0.01	−0.04, 0.07	.666
*Physical neglect*	0.01	−0.05, 0.08	.744
*IPV*	−0.01	−0.08, 0.06	.727
*Household dysfunction*	0.03	−0.03, 0.09	.370
*Age (months)*	−0.05	−0.12, 0.02	.160
*Female gender*	−0.10	−0.21, 0.02	.090
*Chronicity of PA and/or PN*	−0.03	−0.19, 0.13	.688
*SES*	0.06	0.00, 0.12	.**036**
*ADHD*	−0.06	−0.24, 0.12	.498
*Lower education [2]*	−0.44	−0.64, −0.24	**<**.**001**
*Trauma load [2]*	0.02	−0.03, 0.08	.397
**Log-likelihood**	−1683.9		
**ICC**	.05		
**AIC**	3401.9		
**Marginal *R*^2^ / conditional *R*^2^**	.036 / .081		

Note: [2] denotes level 2 predictors. PA: psychological abuse; PN: psychological neglect; SA: sexual abuse; IPV: witnessing domestic violence.

#### 4.5. Sensitivity analysis

Results from the sensitivity analyses showed that using latent-factor scores for PA or PN did not alter the model outcomes. The estimates, significance levels, and model fits remained nearly identical, indicating that the findings were robust to different operationalizations (see Table s12, Supplementary)

## 5. Discussion

This study aimed to improve the understanding of developmental features associated with psychological maltreatment, and to confirm the distinct pathways proposed by the DMAP through which PA and PN may cause harm in a youth general population sample. The DMAP model was only confirmed for the association of threat and dysfunctional emotion regulation. The results indicate that even when considering other relevant predictive factors, PA remained an important predictor of dysfunctional emotion regulation, whereas PN had little explanatory power. In contrast, there was no significant effect of PN on working memory. These differences in explanatory power highlight the distinct harmful characteristics of PA and PN. The DMAP model supported that especially threat was closely linked to dysfunctional emotion regulation. Importantly, ADHD displayed the strongest association with dysfunctional emotion regulation, which underlines additional explanatory factors besides adversity. The trauma load also had a significant impact, highlighting the potential influence of environment on adolescents’ development. In the DMAP_red_, the link of threat and deprivation on dysfunctional emotion regulation aligned, suggesting that their differences in predictive power may partly be driven by PA. PA was clearly associated with dysfunctional emotion regulation, explaining 30% of the variance in the PA-only model and showing a significant effect even when other forms of adversity and deprivation were controlled for. This aligns with previous research (Mills et al., 2015; O’Mahen et al., 2015; Thomassin et al., 2016) and lends support to the assumptions of the DMAP since it suggests that PA disrupts the formation of emotion regulation. Neuroimaging evidence also corroborates this finding, as it has linked PA to neurological alterations in areas and functions potentially associated with emotion processing, such as reduced resting-state functional connectivity in the amygdala and insula (Luo et al., 2022) or reduced hippocampal volume (Carballedo et al., 2012). But alternative explanations are also possible. According to attachment theory, an inappropriate or hostile response by a caregiver can motivate a child to deactivate the manifestation of emotion (Rogier & Velotti, 2023) as they cannot rely on their parents’ positive responsivity. Other psychological mechanisms such as shame or reduced self-compassion, which are reportedly undermined by PA (Ross et al., 2019), may also exacerbate dysfunctional emotion regulation (Paucsik et al., 2023). In addition to the direct effect of PA itself, other more distal influences are also possible, such as intergenerational transmission, which can range from underlying genetic family traits and epigenetic processes (Cecil et al., 2020) to model learning (Assink et al., 2018). Linked to this, household dysfunction was a significant predictor in both the DMAP models and the model including all individual types of adversity.

The association between PN and dysfunctional emotion regulation was not upheld in the full model. Instead, IPV and household dysfunction emerged as significant covariates. This has also been shown in previous studies (i.e. for IPV; Oram et al., 2022) and points to the effect of the familial emotional climate on the emotion regulation of the child (Morris et al., 2007). ADHD and being female emerged as significant covariates, underscoring the pivotal role of individual vulnerability factors in the manifestation of emotion regulation difficulties. These findings further emphasise the importance of considering contextual and individual characteristics in evaluating the impact of PA or psychological maltreatment. The impact of ADHD across all models emphasises that dysfunctional emotion regulation is linked to ADHD (Christiansen et al., 2019), highlighting that there may be other pathways independent of adversity. At the same time, ADHD may act as a risk factor, exacerbating parental difficulties or complicating peer relationships and thereby linking it to abuse, or other conflicts that in turn can lead to dysfunctional emotion regulation. Furthermore, exploratory analyses using subscales instead of (overall) PA as ‘proxy’ predictors showed that Terrorizing/Spurning was most clearly linked to dysfunctional emotion regulation. But it was closely followed by Psychological Control, Overcontrol, and Demanding/Rigid, which are less frequently studied forms of PA. At the same time their harmful consequences have been shown in other research (LeMoyne & Buchanan, 2011; Scharf & Goldner, 2018). Combining all the PA subscales into a single model showed that none of the individual subscales could satisfactorily account for a substantial proportion of the variance.

The DMAP model on working memory failed to support the link between deprivation and working memory. Instead, SES and lower education were significant predictors. Importantly, while these two variables were not included in the deprivation score due to a lack of adequate conceptual fit, they may be linked to an environment with limited resources. Further, the link between PN and working memory was not supported, reflecting inconsistent evidence in the existing literature. This contrasts with studies that have found significant associations, predominantly in high-risk populations (Awada et al., 2025; Sheridan et al., 2017), child-protective-services samples (Demeusy et al., 2018), or institutionalised children (Hanson et al., 2013). There are different possible explanations for this. First, as already noted, many findings stem from studies of institutionalised children exposed to severe deprivation (Lund et al., 2020). In the present study, a measure covering multiple dimensions of deprivation and PN was used to ensure the inclusion of diverse aspects beyond severe cases of deprivation, which may not have a comparable impact on working memory. Second, self-report measures of deprivation and PN are limited to experiences that adolescents recall and report. For cognitive development, a very early deprivation may be especially critical. As discussed by Sheridan et al. (2022), early deprivation may prematurely disrupt neurological processes, even before key developmental periods, and limit ongoing development. The possibility of such early onset and chronic deprivation was not or only partly covered in our measurements. Third, as argued by Spratt et al. (2012), not only the onset of neglect but also its total duration and the subsequent environment are decisive. The majority of students in our sample had spent more than eight years in a well-established school system with structures in place for disadvantaged and at-risk adolescents. Especially for working memory, formal schooling has an impact already starting at primary school, as evidenced by Davidson et al. (2023). This shared educational context of our sample may complicate the attribution of effects to deprivation or PN and highlights the limited generalizability to other samples in different resource contexts.

While these variables were not the focus of the analysis on working memory, and relevant factors such as medication use for ADHD were not included, other expected effects, such as those of the deprivation score, physical neglect, household dysfunction, and ADHD, were not confirmed (Kasper et al., 2012; Lund et al., 2020). Educational level had the greatest explanatory power in the full model. Educational level is shaped not only by intelligence and learning ability but also partly by socioeconomic factors or educational support by parents, which may have some influence on which secondary-school path students are in, and in turn, the educational level may further impact skill development. Educational level is thus somewhat reflective of available resources and determines access to present and future ones, which may contribute to its explanatory power for cognitive outcomes, such as working memory. Higher socioeconomic status also had a small significant positive effect on working memory, pointing to the relevance of available resources.

Overall, these findings are only partially consistent with the DMAP. Specifically, they support the proposed link between threat, and PA more specifically, and dysfunctional emotion regulation, but they do not provide evidence for a similar pathway in the case of PN. As the study did not specifically examine the underlying mechanisms, there are several possible interpretations. First, from a methodological perspective, next to sampling issues, the effects of either deprivation or PN on working memory may not be immediately observable at a single time point. Instead of leading to a set level of working memory, PN might rather influence the developmental trajectory or growth rate of working memory over time (Clinchard et al., 2025). This perspective helps explain why retrospective studies often fail to find a consistent link between PN and working memory (Miskowiak et al., 2023; Tjoelker et al., 2022). Results derived from cross-sectional observation at a time period where executive functioning is still developing (Tervo-Clemmens et al., 2023) may thus fall short in understanding the presumably complex impact of PN on the development of working memory. This effect may have been less pronounced for dysfunctional emotion regulation, given that it was measured via a self-report measure of general perceived emotion regulation abilities. Second, while the presence of threat or PA can shape both psychological and biological processes despite other protective factors, in the case of PN and deprivation, it may be more important whether other sources of support and cognitive stimulation are present. As recent evidence has shown, both peer social support (Glickman et al., 2021) and cognitive reappraisal (Holman & Jignea, 2023) can buffer potential negative effects of PN. Third, our measurement of PN and deprivation may not align clearly with the deprivation dimension proposed in the DMAP, which has been more often operationalised as institutionalisation or lack of stimulation due to poverty. This suggests that PN could represent a qualitatively distinct form of adversity or a form of deprivation that does not neatly fit into the broader category by having other core features leading to different outcomes. Lastly, the DMAP framework may apply more directly to threat/PA, whereas PN may operate through different unidentified mechanisms. In addition, working memory is influenced by various factors beyond adversity including genetics (Miyake & Friedman, 2012). Taken together, these findings underscore the need for a more nuanced understanding of how the core features of PA and PN influence development, while supporting the value of a dimensional perspective as a useful framework.

### 5.1. Limitations

This study features several strengths, including a large sample of adolescents, precise and detailed measurements of PA and PN, and both self-report and performance-task measurements of outcomes. Moreover, a range of important covariates was included and controlled for to ensure the robustness of our findings. Yet there are potential limitations concerning the results of this study. Since we used cross-sectional and retrospective self-report assessments to measure PA and PN, we cannot preclude certain biases, such as the possibility that adolescents assessed their parents’ behaviour depending on their current emotional state and well-being (Danese & Widom, 2020). Due to the low number of non-binary students, these were grouped with the male category, as the predictor of interest was ‘female gender’ because some evidence points to females’ dysfunctional emotion regulation being more susceptible to PA (Hong et al., 2018). Yet this did not allow for separate analysis and may obscure differences between male and non-binary students. Further, a cross-sectional approach does not allow conclusions about causality. Importantly, both outcome variables were measured cross-sectionally at a time when adolescents were presumably still embedded in their family environments. It is essential to consider the contextual nature of both functional and dysfunctional behaviour: Some strategies, such as nonawareness, may be functional in an ongoing abusive environment. Studying them would therefore require a long-term, contextualised perspective that accounts for the adaptive value of certain behaviours in specific environments.

### 5.2. Implications and directions for future research

Focusing on the differential impact of PA and PN improves our understanding of their unique qualities and helps us refine distinct risk groups. Especially youth confronted with PA may profit from interventions targeting the lack of use of dysfunctional emotion regulation strategies. As highlighted in a conceptual review by Villalta et al. (2018), there remains a critical need to deepen our understanding of emotion regulation in youth, particularly with regard to how it can be effectively targeted in diverse populations, including those affected by trauma. Our results on PN partly deviate from previous studies, as working memory performance was not linked to PN but to educational level and socioeconomic status. While it was not possible to test within this sample, a well-established school system where structures are in place to support at-risk students may be effective in counteracting the deleterious effects of PN. PN can thus likely be at least partially compensated for, and establishing secure structures to support disadvantaged and at-risk youth may be crucial. But this finding requires further investigation that considers alternative outcomes and different samples.

## 6. Conclusion

These findings are partially consistent with the DMAP framework since they highlight the significant impact of PA and its uniquely damaging properties on dysfunctional emotion regulation. This direct link may be explained by a variety of mechanisms, including emotional numbing, neurological changes, or transgenerational processes. The hypothesised link between PN and working memory was not supported, and the results suggest that other factors such as socioeconomic status and education level might be more relevant for cognitive development. Thus, PA and PN have distinct associations with developmental features, suggesting that they may pose unique psychopathological risks. Further research is needed to identify their distinct qualities and link these findings to support and intervention strategies.

## Supplementary Material

Supplementary_revised.docx

## Data Availability

The data will be deposited in a publicly accessible repository after the project's end in 2026.
