# Supplementary material for: Understanding the developmental impact of psychological maltreatment through the lens of the dimensional model of adversity and psychopathology
Source: Eur J Psychotraumatol. 2026 Jan 27;17(1):2616980. doi: 10.1080/20008066.2026.2616980 (PMC12849807; doi:10.1080/20008066.2026.2616980)
Supplement: Supplementary_revised.docx [file ZEPT_A_2616980_SM6892.docx]

**Table s1.** Short Description of Items for Each Subscale Included into PA and PN.

|  | **Subscale** | **Short Description** |
| --- | --- | --- |
| **Psychological abuse (PA)** | Demanding/Rigid | Had to be the best |
|  |  | Was very controlling |
|  |  | Used me for emotional needs |
|  |  | Only accepted top grades |
|  | Terrorizing/Spurning | Placed me in frightening situations |
|  |  | Made me cry often |
|  |  | Destroyed my things when angry |
|  |  | Threatened physical harm |
|  |  | Cursed at me |
|  |  | Embarrassed me publicly |
|  | Corrupting | Ignored my wrongdoings |
|  |  | Used illegal drugs with me |
|  |  | Did illegal acts in front |
|  |  | Encouraged illegal acts |
|  | Isolating | Threatened to leave me |
|  |  | Sent me to bed hungry |
|  |  | Punished by confinement |
|  |  | Threatened permanent abandonment |
|  | Psychological Control | Withdrew affection when disagreement |
|  |  | Avoided eye contact if disappointed |
|  |  | Silent treatment until appeased |
|  | Overcontrol | Intrusively monitored my life |
|  |  | Checked my personal belongings |
|  |  | Restricted my friendships |
| **Psychological neglect (PN)** | Emotional Responsiveness (i) | Felt loved by parents |
|  |  | Asked about my day |
|  |  | Showed interest in me |
|  |  | Acknowledged my achievements |
|  |  | Enjoyed spending time with me |
|  |  | Paid attention when I spoke |
|  | Abandonment | Left with unknown people (at a young age) |
|  |  | Left alone for long periods (at a young age) |
|  |  | Thrown out after disagreements |
|  |  | Forgotten about me when I stayed out |
|  | Parental Knowledge (i) | Parents know about free time |
|  |  | Parents know about friends |
|  |  | Parents know about whereabouts at evenings/nights |
|  |  | Parents know about whereabouts after school |

**Table s2.** Correlation Matrix of All Predictors

|  | *Female Gender* | *Chronic PA/PN* | *High Edu [2]* | *Trauma Load [2]* | *PA* | *PN* | *Physical Abuse* | *Intrafam SA* | *IPV* | *Physical Neglect* | *HD* | *SES* | *Age (Months)* | *ADHD* |
| --- | --- | --- | --- | --- | --- | --- | --- | --- | --- | --- | --- | --- | --- | --- |
| **Female Gender** |  |  |  |  |  |  |  |  |  |  |  |  |  |  |
| **Chronic PA/PN** | 0.03 |  |  |  |  |  |  |  |  |  |  |  |  |  |
| **High Edu [2]** | -0.09^**^ | -0.00 |  |  |  |  |  |  |  |  |  |  |  |  |
| **Trauma Load [2]** | 0.01 | 0.12^***^ | 0.43^***^ |  |  |  |  |  |  |  |  |  |  |  |
| **PA** | 0.08^**^ | 0.50^***^ | 0.10^***^ | 0.28^***^ |  |  |  |  |  |  |  |  |  |  |
| **PN** | 0.07^*^ | 0.38^***^ | 0.19^***^ | 0.31^***^ | 0.68^***^ |  |  |  |  |  |  |  |  |  |
| **Physical Abuse** | 0.02 | 0.37^***^ | 0.15^***^ | 0.27^***^ | 0.62^***^ | 0.47^***^ |  |  |  |  |  |  |  |  |
| **Intrafam. SA** | 0.08^**^ | 0.14^***^ | -0.00 | 0.09^**^ | 0.27^***^ | 0.21^***^ | 0.18^***^ |  |  |  |  |  |  |  |
| **IPV** | 0.10^***^ | 0.36^***^ | 0.02 | 0.18^***^ | 0.53^***^ | 0.37^***^ | 0.47^***^ | 0.18^***^ |  |  |  |  |  |  |
| **Physical Neglect** | -0.03 | 0.22^***^ | 0.17^***^ | 0.20^***^ | 0.40^***^ | 0.55^***^ | 0.27^***^ | 0.16^***^ | 0.23^***^ |  |  |  |  |  |
| **HD** | 0.07^*^ | 0.31^***^ | 0.02 | 0.16^***^ | 0.41^***^ | 0.31^***^ | 0.31^***^ | 0.12^***^ | 0.42^***^ | 0.17^***^ |  |  |  |  |
| **SES** | 0.02 | 0.01 | -0.17^***^ | -0.18^***^ | -0.07^*^ | -0.06^*^ | -0.11^***^ | -0.01 | -0.05 | -0.07^*^ | 0.00 |  |  |  |
| **Age (Months)** | 0.03 | 0.04 | 0.02 | 0.30^***^ | 0.09^**^ | 0.11^***^ | 0.09^**^ | 0.02 | 0.09^**^ | 0.03 | 0.09^**^ | -0.05 |  |  |
| **ADHD** | -0.08^**^ | 0.13^***^ | 0.01 | 0.05 | 0.19^***^ | 0.13^***^ | 0.14^***^ | 0.03 | 0.11^***^ | 0.11^***^ | 0.17^***^ | 0.03 | -0.02 |  |
| **Impaired CC** | 0.11^***^ | 0.24^***^ | 0.04 | 0.15^***^ | 0.37^***^ | 0.29^***^ | 0.29^***^ | 0.14^***^ | 0.43^***^ | 0.17^***^ | 0.69^***^ | -0.06^*^ | 0.07^*^ | 0.12^***^ |

*Note*. The correlation was obtained using the Pearson method with listwise deletion. Black denotes significant correlations. [2] denotes level 2 predictors. PA = psychological abuse; PN = psychological neglect; SA = sexual abuse ; IPV = witnessing domestic violence; HD = household dysfunction CC = caregiving conditions. *p < .05. **p < .01.  ***p < .001.

**Table s3.** PA-Only and PN-Only on Dysfunctional Emotion Regulation.

|  | **PA-Only** | | | **PN-Only** | | |
| --- | --- | --- | --- | --- | --- | --- |
| **Predictors** | **Estimate** | **CI** | ***p*** | **Estimate** | **CI** | ***p*** |
| *(Intercept)* | 0.05 | -0.03 – 0.12 | .219 | 0.03 | -0.06 – 0.11 | .512 |
| *PA* | 0.54 | 0.50 – 0.59 | **<.001** | - | - | - |
| *PN* | - | - | - | 0.38 | 0.33 – 0.43 | **<.001** |
| *Age (Months)* | -0.01 | -0.06 – 0.04 | .716 | -0.01 | -0.07 – 0.06 | .859 |
| *Female Gender* | -0.09 | -0.19 – 0.00 | .053 | -0.06 | -0.17 – 0.04 | .249 |
| LogLikelihood | -1497.7 | | | -1614.5 | | |
| ICC | 0.02 | | | 0.03 | | |
| AIC | 3007.4 | | | 3240.9 | | |
| Marginal  *R*^2^ / Conditional *R*^2^ | 0.295 / 0.307 | | | 0.145 / 0.171 | | |

*Note*. PA = psychological abuse; PN = psychological neglect.

**Table s4.** PA-Only and PN-Only on Working Memory.

|  | **PA-Only** | | | **PN-Only** | | |
| --- | --- | --- | --- | --- | --- | --- |
| **Predictors** | **Estimate** | **CI** | ***p*** | **Estimate** | **CI** | ***p*** |
| *(Intercept)* | 0.00 | -0.10 – 0.10 | .965 | 0.00 | -0.10 – 0.10 | .949 |
| *PA* | -0.01 | -0.07 – 0.04 | .628 | - | - | - |
| *PN* | - | - | - | -0.00 | -0.06 – 0.06 | .994 |
| *Age (Months)* | -0.05 | -0.13 – 0.02 | .160 | -0.05 | -0.13 – 0.02 | .153 |
| *Female Gender* | -0.07 | -0.18 – 0.04 | .221 | -0.07 | -0.18 – 0.04 | .206 |
| LogLikelihood | -1697.6  .7 | | | -1697.7 | | |
| ICC | 0.07 | | | 0.07 | | |
| AIC | 3407.2 | | | 3407.5 | | |
| Marginal  *R*^2^ / Conditional *R*^2^ | 0.005 / 0.077 | | | 0.004 / 0.077 | | |

*Note*. PA = psychological abuse; PN = psychological neglect.

**Table s5.** Means and Cronbachs Alpha of PA Subscales

| ***Subscales*** | | ***Mean*** | | ***SD*** | | ***Min*** | | ***Max*** | |  | | ***Cronbach’s Alpha*** | |  | |  | |
| --- | --- | --- | --- | --- | --- | --- | --- | --- | --- | --- | --- | --- | --- | --- | --- | --- | --- |
| **Demanding/Rigid** | 0.83 | | 0.91 | | 0 | | 4.00 | |  | | .79 | |  | |  | |  |
| **Terrorizing/Spurning** | 0.69 | | 0.88 | | 0 | | 4.00 | |  | | .88 | |  | |  | |  |
| **Corrupting** | 0.13 | | 0.34 | | 0 | | 3.25 | |  | | .61 | |  | |  | |  |
| **Isolating** | 0.28 | | 0.62 | | 0 | | 4.00 | |  | | .80 | |  | |  | |  |
| **Psychological Control** | 0.69 | | 0.97 | | 0 | | 4.00 | |  | | .86 | |  | |  | |  |
| **Overcontrol** | 0.53 | | 0.89 | | 0 | | 4.00 | |  | | .86 | |  | |  | |  |

**Table s6.** Explorative Analysis of PA Subscale on Dysfunctional Emotion Regulation.

|  | **Terrorizing/Spurning** | | | **Psychological Control** | | | **Isolating** | | |
| --- | --- | --- | --- | --- | --- | --- | --- | --- | --- |
| **Predictors** | **β** | **95% CI** | ***p*** | **β** | **95% CI** | ***p*** | **β** | **95% CI** | ***p*** |
| *(Intercept)* | 0.13 | -0.07,0.33 | .213 | 0.13 | -0.07,0.34 | .203 | 0.15 | -0.07,0.37 | .182 |
| *Terrorizing/Spurning* | 0.40 | 0.33, 0.48 | **<.001** | - | - | - | - | - | - |
| *Psychological Control* | - | - | - | 0.30 | 0.24,0.36 | **<.001** | - | - | - |
| *Isolating* | - | - | - | - | - | - | 0.08 | 0.00,0.15 | .036 |
| *PN* | 0.09 | 0.02,0.16 | .008 | 0.09 | 0.02,0.16 | .010 | 0.18 | 0.11,0.25 | **<.001** |
| *Physical abuse* | -0.04 | -0.10,0.02 | .200 | 0.02 | -0.04,0.08 | .457 | 0.06 | -0.01,0.12 | .081 |
| *Intrafamilial SA* | 0.00 | -0.05,0.05 | .916 | 0.00 | -0.05,0.05 | .971 | 0.00 | -0.05,0.05 | .870 |
| *Physical neglect* | -0.02 | -0.08,0.03 | .400 | 0.00 | -0.06,0.06 | .982 | -0.01 | -0.06,0.05 | .832 |
| IPV | 0.07 | 0.01,0.12 | .028 | 0.09 | 0.03,0.15 | .004 | 0.12 | 0.06,0.18 | **<.001** |
| *Household Dysfunction* | 0.08 | 0.03,0.14 | .002 | 0.10 | 0.05,0.16 | **<.001** | 0.11 | 0.05,0.16 | **<.001** |
| *Age (Months)* | -0.02 | -0.07,0.04 | .514 | -0.03 | -0.09,0.02 | .247 | -0.03 | -0.09,0.03 | .293 |
| *Female gender* | -0.12 | -0.22,-0.03 | .013 | -0.09 | -0.19,0.00 | .059 | -0.08 | -0.18,0.02 | .127 |
| *Chronic PM* | 0.11 | -0.03,0.24 | .120 | 0.13 | 0.00,0.27 | .056 | 0.23 | 0.09,0.37 | .001 |
| *SES* | 0.02 | -0.02,0.07 | .324 | 0.03 | -0.02,0.08 | .191 | 0.02 | -0.03,0.07 | .436 |
| ADHD | 0.33 | 0.17,0.48 | **<.001** | 0.37 | 0.22,0.53 | **<.001** | 0.38 | 0.22,0.54 | **<.001** |
| *Lower education [2]* | -0.11 | -0.26,0.04 | .167 | -0.13 | -0.28,0.02 | .093 | -0.17 | -0.33,-0.01 | .043 |
| *Trauma Load [2]* | 0.03 | -0.01,0.07 | .179 | 0.04 | 0.00,0.09 | .044 | 0.05 | 0.01,0.10 | .027 |

*Note*. PA = psychological abuse; PN = psychological neglect; SA = sexual abuse ; IPV = witnessing domestic violence.

**Table s7.** Explorative Analysis of PA Subscale on Dysfunctional Emotion Regulation.

|  | **Overcontrol** | | | **Corrupting** | | | **Demanding/Rigid** | | |
| --- | --- | --- | --- | --- | --- | --- | --- | --- | --- |
| **Predictors** | **β** | **95% CI** | ***p*** | **β** | **95% CI** | ***p*** | **β** | **95% CI** | ***p*** |
| *(Intercept)* | 0.16 | -0.05,0.37 | .129 | 0.15 | -0.07,0.36 | .180 | 0.19 | -0.02,0.40 | .077 |
| *Overcontrol* | 0.20 | 0.14,0.26 | **<.001** | - | - | - | **-** | **-** | **-** |
| *Corrupting* | - | - | **-** | 0.05 | -0.01,0.10 | .093 | **-** | **-** | **-** |
| *Demanding/Rigid* | - | - | - | - | - | - | 0.23 | 0.17,0.29 | **<.001** |
| *PN* | 0.14 | 0.07,0.21 | **<.001** | 0.19 | 0.13,0.26 | **<.001** | 0.12 | 0.05,0.19 | .001 |
| *Physical abuse* | 0.04 | -0.02,0.10 | .157 | 0.08 | 0.02,0.14 | .013 | 0.05 | -0.01,0.11 | .118 |
| *Intrafamilial SA* | -0.01 | -0.06,0.04 | .722 | 0.01 | -0.04,0.06 | .749 | 0.00 | -0.04,0.05 | .859 |
| *Physical neglect* | 0.00 | -0.06,0.05 | .920 | -0.01 | -0.07,0.05 | .748 | -0.01 | -0.06,0.05 | .844 |
| *IPV* | 0.11 | 0.05,0.17 | **<.001** | 0.13 | 0.07,0.19 | **<.001** | 0.12 | 0.06,0.18 | **<.001** |
| *Household Dysfunction* | 0.10 | 0.04,0.15 | **<.001** | 0.11 | 0.06,0.17 | **<.001** | 0.10 | 0.05,0.16 | **<.001** |
| *Age (Months)* | -0.03 | -0.08,0.03 | .356 | -0.04 | -0.09,0.02 | .229 | -0.04 | -0.10,0.01 | .145 |
| *Female gender* | -0.09 | -0.18,0.01 | .084 | -0.07 | -0.17,0.03 | .174 | -0.07 | -0.17,0.02 | .133 |
| *Chronic PM* | 0.20 | 0.06,0.34 | .005 | 0.24 | 0.10,0.38 | .001 | 0.16 | 0.02,0.29 | .026 |
| *SES* | 0.03 | -0.02,0.08 | .318 | 0.02 | -0.03,0.07 | .402 | 0.03 | -0.02,0.08 | .294 |
| ADHD | 0.36 | 0.21,0.52 | **<.001** | 0.39 | 0.23,0.54 | **<.001** | 0.38 | 0.22,0.54 | **<.001** |
| *Lower education [2]* | -0.17 | -0.32,-0.01 | .035 | -0.17 | -0.33,-0.01 | .036 | -0.19 | -0.35,-0.03 | .017 |
| *Trauma Load [2]* | 0.05 | 0.00,0.09 | .030 | 0.05 | 0.01,0.10 | .027 | 0.05 | 0.01,0.10 | .020 |

*Note*. PA = psychological abuse; PN = psychological neglect; SA = sexual abuse ; IPV = witnessing domestic violence.

| **Model** | **AIC** | **BIC** | **LogLik** | **Marginal R^2^** | **Conditional R^2^** |
| --- | --- | --- | --- | --- | --- |
| *Demanding/Rigid* | 3043.83 | 3130.46 | -1504.91 | 0.29 | 0.30 |
| *Terrorizing/Spurning* | 2988.03 | 3074.66 | -1477.02 | 0.32 | 0.33 |
| *Corrupting* | 3099.26 | 3185.89 | -1532.63 | 0.26 | 0.27 |
| *Isolating* | 3097.70 | 3184.33 | -1531.85 | 0.26 | 0.27 |
| *Psychological Control* | 3022.11 | 3108.74 | -1494.06 | 0.30 | 0.31 |
| *Overcontrol* | 3061.28 | 3147.91 | -1513.64 | 0.28 | 0.29 |

**Table s8.** Model Fit Statistics for PA Subscales on Dysfunctional Emotion Regulation.

**Table s9.** Model Fit and Partial R^2^ for Full Model including all PA Subscales

| **Model Statistics** | **AIC** | **BIC** | **LogLik** | **Marginal R^2^** | **Conditional R^2^** |
| --- | --- | --- | --- | --- | --- |
| *Full Model* | 2958.44 | 3070.55 | -1457.22 | 0.34 | 0.35 |
| **PA Subscales** | **Partial R^2^** | **95% CI** |  |  |  |
| *Corrupting* | 0.00 | 0.00, 0.02 |  |  |  |
| *Demanding/Rigid* | 0.00 | 0.00, 0.02 |  |  |  |
| *Isolating* | 0.00 | 0.00, 0.00 |  |  |  |
| *Overcontrol* | 0.00 | 0.00, 0.00 |  |  |  |
| *Psychological Control* | 0.01 | 0.00, 0.03 |  |  |  |
| *Terrorizing/Spurning* | 0.02 | 0.00 - 0.04 |  |  |  |

**Table s10.** Full and Reduced DMAP Model on Emotional Dysregulation.

|  | **Full DMAP Model** | | | **Reduced DMAP Model** | | |
| --- | --- | --- | --- | --- | --- | --- |
| **Predictors** | **β** | **95% CI** | ***p*** | **β** | **95% CI** | ***p*** |
| *(Intercept)* | 0.03 | -0.06 – 0.11 | .535 | 0.02 | -0.07 – 0.11 | .646 |
| *Threat- Score* | 0.34 | 0.27 – 0.40 | <.001 | - | - | - |
| *Threat- Score (Reduced)* | - | - | **-** | 0.21 | 0.15 – 0.27 | <.001 |
| *Deprivation- Score* | 0.09 | 0.03 – 0.16 | .006 | 0.18 | 0.11 – 0.24 | <.001 |
| *Age (Months)* | -0.03 | -0.09 – 0.03 | .304 | -0.03 | -0.09 – 0.03 | .306 |
| *Female gender* | -0.08 | -0.18 – 0.01 | .092 | -0.07 | -0.17 – 0.03 | .161 |
| *SES* | 0.03 | -0.02 – 0.08 | .214 | 0.03 | -0.02 – 0.08 | .292 |
| *Household Dysfunction* | 0.09 | 0.03 – 0.15 | .002 | 0.11 | 0.05 – 0.17 | <.001 |
| *ADHD* | 0.39 | 0.23 – 0.55 | <.001 | 0.43 | 0.26 – 0.59 | <.001 |
| *Lower Education [2]* | -0.16 | -0.32 – 0.00 | .053 | -0.18 | -0.34 – -0.02 | .031 |
| *Trauma Load [2]* | 0.05 | 0.01 – 0.09 | .028 | 0.06 | 0.02 – 0.11 | .008 |
| LogLikelihood | -1530.358 | | | -1559.920 | | |
| ICC | 0.02 | | | 0.02 | | |
| AIC | 3084.715 | | | 3143.840 | | |
| Marginal R2 / Conditional R2 | 0.258 / 0.274 | | | 0.222 / 0.240 | | |

**Table s11.** Full and Reduced DMAP Model on Working Memory

|  | **Full DMAP Model** | | | | **Reduced DMAP Model** | | | |  |
| --- | --- | --- | --- | --- | --- | --- | --- | --- | --- |
| **Predictors** | **β** | | **95% CI** | ***p*** | **β** | | **95% CI** | ***p*** |  |
| *(Intercept)* | 0.12 | | 0.02 – 0.23 | .025 | 0.12 | | 0.01 – 0.23 | .026 |  |
| *Threat- Score* | -0.03 | | -0.10 – 0.04 | .375 | -0.03 | | -0.09 – 0.04 | .458 |  |
| *Deprivation- Score* | 0.03 | | -0.05 – 0.11 | .436 | - | | - | - |  |
| *Deprivation- Score (Reduced)* | - | | - | - | 0.02 | | -0.05 – 0.09 | .564 |  |
| *Age (Months)* | -0.05 | | -0.12 – 0.02 | .160 | -0.05 | | -0.12 – 0.02 | .163 |  |
| *Female gender* | -0.09 | | -0.21 – 0.02 | .099 | -0.09 | | -0.21 – 0.02 | .102 |  |
| *SES* | 0.06 | | 0.01 – 0.12 | .031 | 0.06 | | 0.01 – 0.12 | .030 |  |
| *Household Dysfunction* | 0.02 | | -0.05 – 0.08 | .623 | 0.02 | | -0.05 – 0.09 | .627 |  |
| *ADHD* | -0.07 | | -0.25 – 0.11 | .469 | -0.07 | | -0.25 – 0.12 | .478 |  |
| *Lower Education [2]* | -0.44 | | -0.64 – -0.24 | <.001 | -0.43 | | -0.63 – -0.23 | <.001 |  |
| *Trauma Load [2]* | 0.02 | | -0.03 – 0.08 | .397 | 0.02 | | -0.03 – 0.08 | .376 |  |
| LogLikelihood | | -1684.571 | | | | -1684.691 | | | |
| ICC | | 0.05 | | | | 0.05 | | | |
| AIC | | 3393.141 | | | | 3393.383 | | | |
| Marginal  *R*^2^ / Conditional *R*^2^ | | 0.035 / 0.080 | | | | 0.035 / 0.079 | | | |

**Table s12.** Sensitivity Analysis for Full Models.

|  | **Full Model Emotional Dysregulation** | | | **Full Model Working Memory** | | |
| --- | --- | --- | --- | --- | --- | --- |
| **Predictors** | **β** | **95% CI** | ***p*** | **β** | **95% CI** | ***p*** |
| *(Intercept)* | 0.15 | -0.05 – 0.34 | .139 | 0.56 | 0.30 – 0.83 | <.001 |
| ***PA as Latent Factor Score*** | 0.47 | 0.39 – 0.55 | <.001 | -0.01 | -0.10 – 0.09 | .898 |
| ***PN as Latent Factor Score*** | 0.03 | -0.04 – 0.10 | .337 | 0.03 | -0.07 – 0.13 | .531 |
| *Physical abuse* | -0.04 | -0.10 – 0.02 | .193 | -0.03 | -0.11 – 0.04 | .374 |
| *Intrafamilial SA* | -0.02 | -0.07 – 0.03 | .434 | 0.01 | -0.04 – 0.07 | .667 |
| *Physical neglect* | -0.01 | -0.07 – 0.04 | .677 | 0.01 | -0.06 – 0.08 | .751 |
| *IPV* | 0.06 | 0.00 – 0.12 | .036 | -0.01 | -0.08 – 0.06 | .725 |
| *Household Dysfunction* | 0.08 | 0.03 – 0.14 | .002 | 0.03 | -0.03 – 0.09 | .371 |
| *Age (Months)* | -0.02 | -0.08 – 0.03 | .379 | -0.05 | -0.12 – 0.02 | .161 |
| *Female gender* | -0.11 | -0.21 – -0.02 | .022 | -0.10 | -0.21 – 0.01 | .088 |
| *Chronic PM* | 0.07 | -0.06 – 0.21 | .288 | -0.03 | -0.19 – 0.13 | .686 |
| *SES* | 0.03 | -0.02 – 0.08 | .212 | 0.06 | 0.00 – 0.12 | .036 |
| ADHD | 0.33 | 0.18 – 0.48 | <.001 | -0.06 | -0.24 – 0.12 | .504 |
| *Lower education [2]* | -0.12 | -0.27 – 0.02 | .103 | -0.44 | -0.64 – -0.24 | <.001 |
| *Trauma Load [2]* | 0.04 | -0.00 – 0.08 | .179 | 0.02 | -0.03 – 0.08 | .399 |
| LogLikelihood | -1474.067  .7 | | | -1683.922  .7 | | |
| ICC | 0.01 | | | 0.04 | | |
| AIC | 2982.134 | | | 3401.845 | | |
| Marginal  *R*^2^ / Conditional *R*^2^ | .326 / .335 | | | .036 / .081 | | |

*Note*. PA = psychological abuse; PN = psychological neglect; SA = sexual abuse ; IPV = witnessing domestic violence. The latent PA score was constructed identically to the existing second-order CFA ([https://doi.org/10.1016/j.chiabu.2025.107544).](https://doi.org/10.1016/j.chiabu.2025.107544).%20For%20the%20latent%20 ) For the latent PN score, parentification was replaced by the parental knowledge scale, on which all items loaded above .7. The three PN subscales showed adequate loadings on the second-order PN factor: Emotional Responsiveness (.84), Parental Knowledge (.60), and Abandonment (.69).
